# Dose-tapering trajectories in patients with remitted psychosis undergoing guided antipsychotic reduction to reach minimum effective dose

**DOI:** 10.1192/j.eurpsy.2023.2440

**Published:** 2023-08-14

**Authors:** Chen-Chung Liu, Ming H. Hsieh, Yi-Ling Chien, Chih-Min Liu, Yi-Ting Lin, Tzung-Jeng Hwang, Hai-Gwo Hwu

**Affiliations:** 1 Department of Psychiatry, National Taiwan University Hospital, Taipei, Taiwan; 2 Department of Psychiatry, College of Medicine, National Taiwan University, Taipei, Taiwan

**Keywords:** antipsychotics, minimum effective dose, quality of life, tapering, trajectory

## Abstract

**Background:**

Patients with remitted psychosis wish to reduce antipsychotic doses yet facing increased risks of relapse. Examining dose-tapering processes may provide insights to re-evaluate the risk-to-benefit balance. We aimed to depict and subgroup tapering trajectories, and explore factors associated with different dose-reduction patterns.

**Methods:**

A 2-year open-label randomized prospective comparative trial from August 2017 to September 2022 in Taiwan. Patients with a history of schizophrenia-related psychotic disorders under stable medications and symptoms were eligible, randomizing a proportion to conduct guided dose reduction. We depicted the trajectories of individual patients and named subgroups based on dose-tapering patterns. Predictors of baseline characteristics for designated subgroups were examined by logistic regression analysis; changes in outcomes were compared by paired t-test.

**Results:**

Fifty-one patients undergoing guided dose reduction, 18 (35.3%) reduced 4 steps consecutively (sequential reducers, SR), 14 (27.5%) reduced 1 to 3 steps (modest reducers, MR), 3 (5.9%) re-escalated to previous level (alert reducers, AR), 7 (13.7%) returned to baseline level (baseline returners, BR), 6 (11.7%) relapsed (failed reducers, FR) and 3 (5.9%) withdrew without relapse (early exits, EE). Patients with a history of relapse assumed a conservative dose-tapering pace; only the SR subgroup exhibited significant improvements in functioning and quality of life while failing to identify variables for predicting who would become SR or FR.

**Conclusions:**

Guided dose reduction comprises dynamic processes with differences between individual trajectories. The proposed naming of dose-tapering patterns/subgroups provides a framework depicting patients undergoing dose-tapering. Longer-term observation and more flexible tapering approaches are anticipated to reveal favorable outcomes.

## Introduction

Patients with schizophrenia are inclined to discontinue antipsychotics once remission is achieved for a variety of reasons [1], but current evidence does not favor this discontinuance due to concerns about the higher risk of relapse [2] and poorer prognosis once relapse has occurred [3, 4]. While valid predictors to identify individuals who can discontinue medication successfully are scarce [5, 6], clinicians’ attitudes towards discontinuing antipsychotics in patients with remitted psychosis are a mix of anticipation and apprehension [7–9]. The debates continue between maintenance versus discontinuation of antipsychotics for stable patients because no consensus has been reached while weighing the detrimental results of relapse following medication discontinuation [2, 10, 11], and the potential hazards of long-term antipsychotic treatment on patient’s physical health, cognitive functioning, and even brain structure [12–15].

Dose-reduction was expected to be a compromise solution for this debate [16], and a few studies have reported findings supporting this approach [17–19]. The dose-reduction trials of Uchida et al. found that the therapeutic window of D2 receptor blockade could be lower than 65% for stable patients [20–22]; accordingly, the dose during maintenance should be lower than the commonly recommended levels. Even though a meta-analysis suggested that the risk of relapse in patients undergoing dose reduction is twofold of their maintenance counterparts, neurocognitive functioning in the former is significantly improved after dose reduction [23]. A recent systematic review reported similar findings and called for more attention to this approach concerning its potential to improve patients’ quality of life and global functioning [24].

While guidelines advise that patients may try to discontinue antipsychotics once they have been remitted from a first psychotic episode [25], no guideline has explicitly addressed how to achieve this goal safely. A few longitudinal studies have presented favorable long-term outcomes of patients who remained drug-free, clinically stable, and with good functioning [26–28], yet they have provided little information regarding how to reach such a favorable status. On the other hand, several medication discontinuation trials that endorsed maintenance therapy may have overestimated the risk of relapse, either discontinuing antipsychotics too fast, which rendered patients at risk of dopamine supersensitivity psychosis [29], or monitoring symptoms too closely after medication discontinuation, resulting in misrecognizing symptoms associated with antipsychotic withdrawal as a form of relapse [30]. Thus, a closer look at dose tapering is needed to examine the underpinnings.

The pioneering work of Wunderink *et al.* regarding guided antipsychotic discontinuation revealed that 46.2% of subjects did not completely stop antipsychotics within 18 months; among those who had once reached discontinuation, only 21.5% remained in a remitted state, 24.6% restarted antipsychotics due to relapse, and 7.7% restarted medication once mild recurrent symptoms re-emerged [31]. In a small pilot study of patients with remitted chronic psychosis, Huhn et al. illustrated detailed 6-month trajectories of individuals undergoing dose tapering [19]. Gaebel et al. used graphic presentations, including relevant clinical information, in representative case vignettes to depict long-term treatment with antipsychotics [32]. Their works provide a framework by which to exemplify the dynamic processes during dose-tapering, which is pivotal in developing a guided dose-reduction algorithm to reach a desirable and balanced risk-to-benefit ratio.

Several clinical trials were initiated to test whether or not it is feasible to conduct medication reduction/discontinuation for better functioning outcomes [33–36]. Likewise, our team conducted a prospective comparative cohort study from August 2017 to September 2022 in Taiwan to test the protocol of guided antipsychotic reduction to reach minimum effective dose (GARMED) [37]. Different from previous trials which aimed to discontinue antipsychotics completely while accepting an increased risk of relapse as the tradeoff, the goal of GARMED was to challenge whether stable patients could successfully reduce doses without increased risk of relapse. As shown by our previous observations in a naturalistic cohort [38], we expected the present trial to reach a dose much lower than the minimum effective dose (MED) estimated by doses that exert a distinguishable improvement in symptoms shown in randomized, double-blind, fixed-dose trials comparing antipsychotics to placebo [39, 40].

Currently, our GARMED trial revealed that the relapse rate of the guided dose reduction (GDR) group was not higher than their maintenance counterparts [41]. Among the 51 GDR patients in this study, 74.5% stayed well and relapse-free under a dose lower than their baseline level, while not all of them could reduce doses successively as planned during a 2-year observation-GDR trial. On the other hand, 25.5% of GDR patients were not able to reduce any dose, either having a relapse or having to resume their baseline dose to prevent relapse during dose tapering. In this report, we focused on examining GDR patients with specific interests to (1) depict and subgroup individual trajectories during the course of dose reduction, (2) compare baseline demographic and clinical characteristics between patients with different trajectories, and (3) explore factors associated with dose tapering patterns.

## Methods

### Design, setting, and participants

**Figure 1.**
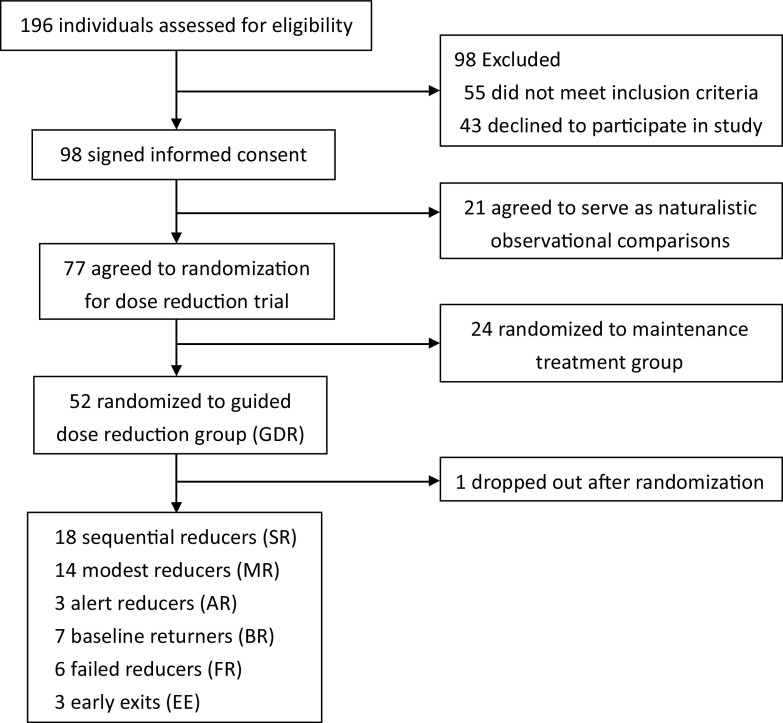
Diagram of trial flow chart.

This open-label randomized prospective comparative cohort trial was based on a previously described pragmatic design (Figure 1) [37]. In brief, patients with remitted psychosis (a history of schizophrenia-related psychotic disorders based on the Diagnostic and Statistical Manual of Mental Disorders, Fifth Edition [DSM-5] diagnosis) under treatment with a stable antipsychotic dose over 3 months were eligible (see eligible criteria in Supplementary Table S1). After being briefed with a psychoeducation session regarding the rationale for this GDR trial and possible risks and benefits during tapering, two-thirds of eligible patients willing to try guide dose reduction were randomized to GDR group. All included patients provided signed informed consent to participate before starting the trial. Patients younger than age 20 years provided signed consent from their parents or guardians in accordance with Taiwan law. Ethical approval was obtained from the Research Ethics Committee of the study hospital (REC: 201703002RIND), and this study was registered at ClinicalTrials.gov (identifier: NCT03248180).

### Dose-reduction process

The rationale and details of the dose-reduction algorithm were also detailed in previous papers [16, 37]. In brief, the extent of dose reduction is small (reducing no more than one-quarter of the current dose at a time), and the tempo of tapering is slow (the next dose reduction attempt would not be initiated until 24 weeks of stabilization). In addition, patients needed to be monitored every 4 weeks for three consecutive sessions once undergoing dose reduction, and they would stay at the same dose for another 12 weeks if maintained stabilized, defined as no aggravation of symptoms lasting for more than 1 week during this time span.

Importantly, patients received instructions regarding suspected symptoms of relapse during tapering, the possibility of a need to resume a rescue dose, and how to call for help when not sure about their subjective changes. In such cases, extra return visits were arranged and patients were supervised to resume doses at the previous level if experiencing suspected signs of relapse. If any one of the 3 positive symptoms and 2 general symptoms in the inclusion criteria could not be stabilized within 1 week (i.e., scores >3) under their baseline antipsychotic dose, the patient would be treated with a higher dose and designated as having a relapse. If maintained stable under a reduced dose for 24 weeks, patients were empowered with a shared decision-making process regarding whether they wished to try another dose reduction or not. Patients could decide to stay at the current dose for a more extended time if feeling not ready for further dose tapering at the designated timepoint.

The subsequent dose reduction was a reiteration of the previous step, cutting off one-quarter of the current dose, yielding 9/16 (3/4 × 3/4) of the baseline dose (in a hyperbolic manner), rather than cutting off another 1/4 of the baseline dose (in a linear manner), as the latter would result in reducing 1/2 (1/4 + 1/4) of the baseline dose after two dose-reductions and end at zero by employing four dose reducing steps, while the first formula, (3/4)^n^, is able to be *re-iterated indefinitely*—a strategy feasible for minimizing the negative impact of dopamine-supersensitivity [42]. Also, the actual daily doses taken during tapering would not always fit precisely with the numbers calculated by this formula. The latter just served as the minimum dose for each timepoint since it was impractical to cut off a quarter or even smaller piece of a tablet for daily dosing [37].

### Depicting and subgrouping trajectories of dose tapering

To illustrate individual trajectories of dose tapering in a consistent manner, the doses at each timepoint of individual patient were first transformed as the proportions of their baseline doses; that is, the baseline level was designated as “1”, uniformly across all patients, regardless of which antipsychotics and doses were received at the beginning of this trial. Second, the current dose/baseline dose was calculated to make up plots comprising all fractions of doses at each timepoint of the individual patients, in order to portray a constellation of trajectories for all patients using a standardized visual presentation. Then, the patterns of patients could be visually identified straightforwardly based on their trajectories during dose tapering, as follows:

1) *Sequential reducer* (SR): Applied to those who tapered doses successfully at each designated timepoint for 4 steps in 2 years; 2) *Modest reducer* (MR): Applied to those who took a cautious pace in tapering, only reducing 1 to 3 steps, with no re-escalation to previous steps, during 2 years; 3) *Alert reducer* (AR): Applied to those who reduced at least 2 steps yet re-escalated the dose back to the level between their baseline dose and the lowest dose reached during the dose-tapering course; 4) *Baseline returner* (BR): Applied to those who tapered at least 1 step but returned to the baseline dose due to concern about risk of relapse and were able to stay in remission throughout the course; 5) *Failed reducer* (FR): Applied to those who had a relapse during dose tapering and were in need of a dose higher than their baseline level; 6) *Early exit* (EE): Applied to those who withdrew from the trial after tapering at least 1 step without relapse before the end of 2-year follow-up.

**Figure 2.**
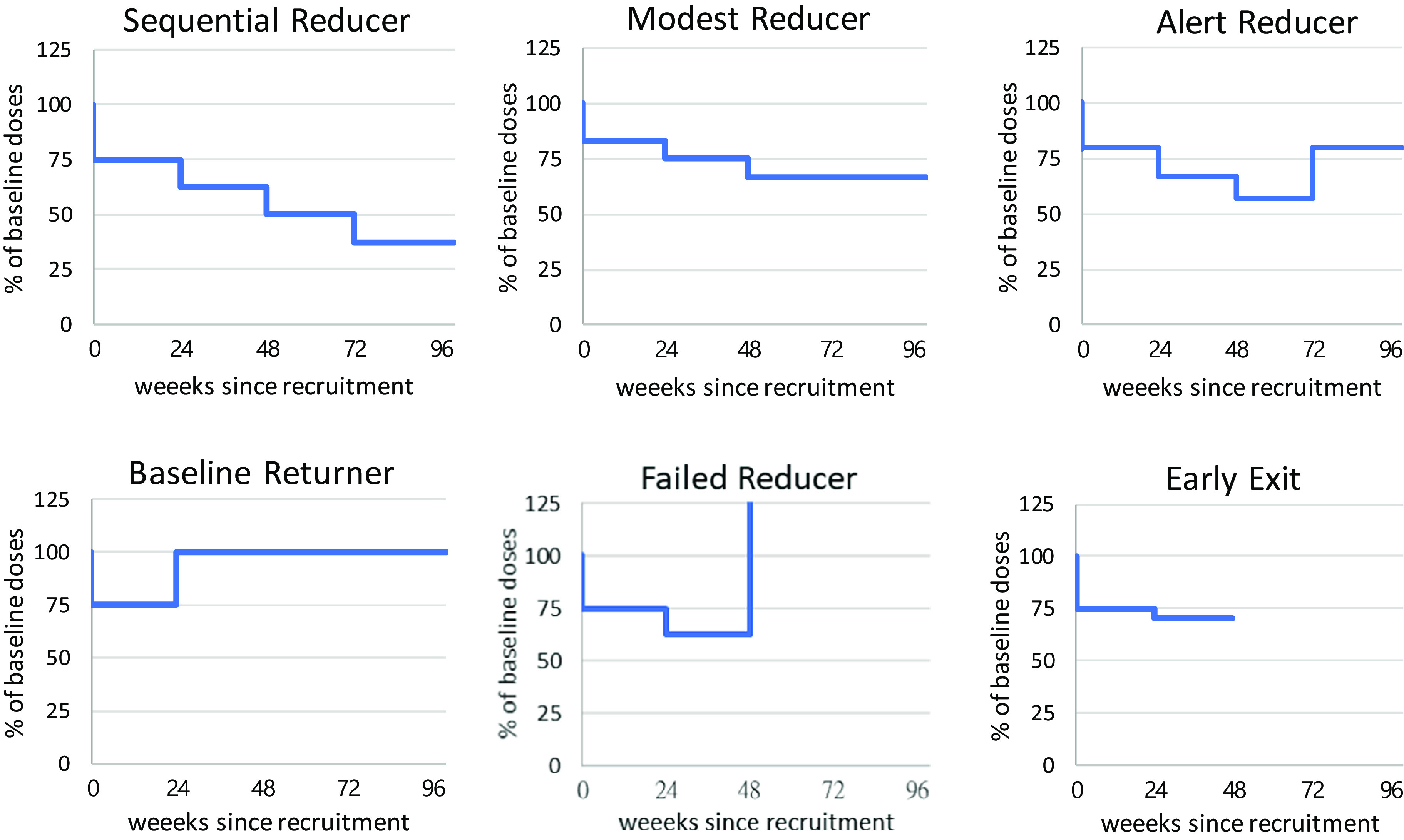
Patterns of dose tapering trajectories. Sequential reducers (SR), those who were able to taper doses at each designated timepoint successfully for four steps in 2 years; Modest reducer (MR), those who took a cautious pace in tapering, thus they only reduced 1 to 3 steps, with no re-escalation to previous step, during 2 years; Alert reducer (AR), those who have had reduced at least two steps yet re-escalated dose back to the level between their baseline dose and the lowest dose they have reached during the course of dose tapering; Baseline returner (BR), those who have tapered at least 1 step but returned to baseline dose for concern about risks of relapse and were able to stay in remission throughout the course; Failed reducer (FR), those who had a relapse during dose tapering and were in need of a dose higher than their baseline level; Early exit (EE), those who left the trial after tapering at least 1 step without relapse before the end of 2-year follow-up. AR, BR, and MR were collapsed as conservative reducers (CR) in further analyses.

Examples of subjects with each pattern are illustrated in Figure 2. Constellations of all individual patient’s trajectories are presented in Supplementary Figure S1. The modest reducers, alert reducers, and baseline returners were further collapsed as a subgroup of “conservative reducers (CR)” to be compared with the other 3 subgroups.

### Clinical assessments

Clinical severities were rated using the Mandarin version Positive and Negative Syndrome Scale (PANSS) [43], Clinical Global Impression of Severity (CGI-S), and Personal and Social Performance scale (PSP) [44], which were carried out by each patient’s attending psychiatrist. Patients’ employment status was collected by face-to-face interview with our research assistant. Patients’ subjective experiences were reported by filling out a medication satisfaction questionnaire (MSQ) based on a 7-point Likert scale [45], and using the EuroQoL-5D visual analogue scale (EQ-5D-VAS) to evaluate quality of life [46]. In addition, each patient’s medical records were reviewed to collect demographics, clinical diagnosis, age of onset, illness duration, and any history of psychiatric hospitalization and psychotic relapse during their illness.

### Statistics

To examine between-group differences, baseline clinical characteristics were compared between groups using χ^2^ tests for categorical variables and analyses of variance (ANOVAs) for continuous variables. Differences in scores between baseline and 2-year follow-up were compared using the paired *t*-test. To explore whether the patient with certain factors would more likely become a sequential reducer, conservative reducer, or failed reducer, univariate logistic regression followed by multivariate logistic regression analysis with backward stepwise selection of baseline clinical variables, and eliminating variables with a probability >0.2, were employed, respectively. Hypothesis testing and confidence intervals were two-sided at the 5% significance level (STATA 13; Stata Corp., College Station, Texas, USA).

## Results

### Baseline characteristics

The diagram of this trial is illustrated in Figure 1. Among the 51 patients assigned to guided dose-reduction group, there were 18 sequential reducers, 14 modest reducers, 3 alert reducers, 7 baseline returners, 6 failed reducers, and 3 early exits. The details of baseline demographic and clinical characteristics among 6 patterns of trajectories are presented in Table 1. Comparisons of demographic variables and changes between baseline and end-of-follow-up clinical variables are displayed in Table 2.

**Table 1.** Baseline demographic and clinical characteristics of participants among 6 trajectories

	SR (*n* = 18)	MR (*n* = 14)	AR (*n* = 3)	BR(*n* = 7)	FR(*n* = 6)	EE(*n* = 3)	Total(*n* = 51)
Age, Years (SD)	35.6 (10.7)	36.8. (8.6)	34.3 (5.7)	35.1 (7.8)	27.5 (9.0)	26.3(11.2)	34.3 (9.6)
Sex, Male (%))	11 (61.1%)	4 (28.6%)	2 (66.7%)	3 (42.9%)	3 (50%)	3 (100%)	26 (51%)
Employment ^a^							
Full-time	7	3	1	3	3	1	18
Part-time	8	7	1	2	1	0	19
No	3	4	1	2	2	2	14
Diagnosis ^b^							
Schizophrenia	12	13	3	6	6	1	41
Other psychotic disorders	6	1	0	1	0	2	10
Duration of illness, years (SD)	10.7 (11.1)	10.9 (7.8)	7 (4.6)	10.3 (4.0)	5.2 (6.4)	6.9 (8.8)	9.6 (8.5)
History of hospitalization (%)	11 (61.1%)	6 (42.9%)	1 (33.3%)	5 (71.3%)	2 (33.3%)	2 (66.7%)	29 (56.9%)
History of relapse (%)	12 (66.7%)	12 (85.7%)	2 (66.7%)	6 (85.7%)	2 (33.3%)	0 (0%)	34 (66.7%)
CPZE mg/d, (SD)	212.9 (171.2)	215.4 (112.7)	195.8 (28.7)	191.1 (84.4)	147.9 (88.8)	293.3 (229.4)	206.7 (134.1)
PANSS total score (SD)	37.6 (5.8)	40.4 (4.5)	46.3 (4.7)	35.7 (4.3)	48.3 (21.1)	48.7 (10.1)	40.5 (9.7)
CGI-S score (SD)	2.0 (0.84)	2.14 (0.77)	2.33 (0.58)	1.43 (0.53)	2.0 (0.89)	3 (0)	2.04 (0.8)
PSP score (SD)	81.4 (7.0)	79.5 (6.8)	77.7 (5.9)	84.1 (4.7)	78.8 (15.0)	70.3 (2.1)	80.1 (8.4)
EQ-5D-VAS (SD)	76.8 (13.4)	72.1 (15.3)	77 (7)	73.6 (9.4)	80.5 (12.9)	70 (10)	75.1 (12.8)
MSQ score (SD)	5.17 (1.58)	5.36 (1.22)	6 (1)	5.14 (0.9)	5.5 (1.05)	4.67 (1.15)	5.27 (1.27)
Antipsychotics							
Amisulpride	1	1	–	1	–	–	3
Aripiprazole-oral	4	4	–	3	2	1	14
Aripiprazole-LAI	–	–	1	–	–	–	1
Clozapine	1	2	–	–	–	–	3
Lurasidone	2	1	–	–	–	–	3
Olanzapine	4	1	–	2	1	–	8
Paliperidone-oral	1	2	–	1	–	1	5
Paliperidone-LAI	–	–	1	–	–	–	1
Quetiapine	1	–	–	–	–	–	1
Risperidone	2	2	1	–	2	1	8
Sulpiride	2	–	–	–	1	–	3
Ziprasidone	–	1	–	–	–	–	1

Abbreviations: *Naming subgroups*: AR: alert reducer; BR: baseline returner; EE: early exit from follow-up; FR: failed reducer; MR: modest reducer; SR: sequential reducer; *Baseline and clinical variables*: CGI-S: clinical global impression-severity; CPZE: chlorpromazine equivalent dose; EQ-5D-VAS: EuroQoL-5D visual analogue scale; LAI: long-acting injectable; MSQ: medication satisfaction questionnaire; PANSS: positive and negative syndrome scale; PSP: personal and social performance.

a Full-time employment defined as having a job >15 h per week for more than 3 months during the past 6 months; Part-time employment defined as serving some duties or having a job but not meeting the requirement for full-time employment.

b Schizophrenia included schizophrenia and schizophreniform disorder; Other psychotic disorder included other schizophrenia-spectrum and other psychotic disorder based on DSM-5 criteria.

**Table 2.** Comparisons of demographic/clinical characteristics and changes between baseline and 2-year follow-up across 4 subgroups

	SR (*n* = 18)	CR (*n* = 24)	FR (*n* = 6)	EE (*n* = 3)	Total (*n* = 51)	*p* value^a^
Diagnosis,^b^ schizophrenia (%), BL	12 (66.7%)	22 (91.7%)	6 (100%)	1 (33.3%)	41 (80.4%)	**0.029**
History of relapse (%), BL	12 (66.7%)	20 (83.3%)	2 (33.3%)	0 (0%)	34 (66.7%)	**0.007**
PANSS total score (SD), BL	37.6 (5.8)	39.7 (6.5)	48.3 (21.1)	48.7 (10.1)	40.5 (9.7)	**0.045**
Full-time Employed,^c^ Yes (%), BL	7 (38.95)	7 (29.2%)	3 (50%)	1 (33.3%)	18 (35.3%)	0.611
Full-time Employed,^c^ Yes (%), FU	12 (66.7%) ^d^	9 (37.5%)	0 (0%)	1 (33.3%)	22 (43.1%)	**0.020**
∆ CPZE mg/d, (SE)	−123.3 (22.5) ^d^	−57.3 (11.8) ^d^	^e^	−57.2 (30.1)		
∆ PANSS total score (SE)	−3.94 (1.25) ^d^	0.43 (1.09)	20.33 (9.12)^f^	11.33 (4.1)		
∆ CGI-S score (SE)	−0.44 (0.15) ^d^	−0.08 (0.08)	2 (0.58)	−0.33 (0.33)		
∆ PSP score (SE)	4.6 (1.3) ^d^	1.4 (0.8)	−16.8 (8.4) ^f^	3 (1.7)		
∆ EQ-5D-VAS (SE)	7 (2.55) ^d^	5.38 (3.4)	−10.5 (4.35)	0 (0)		
∆ MSQ score (SE)	0.38 (0.28)	0.17 (0.18)	^g^	0.33 (1.45)		

Abbreviations: *Subgroup naming:* SR: sequential reducer; CR: conservative reducer (comprising MR modest reducer, AR alert reducer, and BR baseline returner); FR: failed reducer; EE: early exit from follow-up; *Baseline and clinical variables:* BL: baseline; CGI-S: clinical global impression-severity; CPZE chlorpromazine equivalent dose; ∆: within-subgroup differences between the end of follow-up and baseline examined by paired *t*-test; EQ-5D-VAS: EuroQoL-5D visual analog scale; FU: end of follow-up status; PANSS: positive and negative syndrome scale; PSP: personal and social performance; SD: standard deviation; SE: standard error.

a *p* value denotes the statistics among 4 subgroups.

b Schizophrenia includes schizophrenia and schizophreniform disorder, versus other schizophrenia-spectrum and other psychotic disorders.

c Full-time employment is defined as having a job >15 hours per week for more than 3 months during the past 6 months.

d Within a subgroup, the end-of-follow-up scores were statistically significantly different from their baseline scores compared to paired t-test.

e Two patients actually stopped medications on their own accord, the other four patients had relapsed and even resumed a dose higher than the baseline level.

f Excluding a case who started from exceptionally high baseline PANSS scores and then steadily decreased severity during the course until relapse, the remaining 5 failed reducers showed significant worsening in PANSS and PSP comparing their scores at baseline and at the time of relapse by paired t-test.

g Patients having a relapse did not report MSQ by that time. Statistics with *p* values < 0.05 were displayed in bold.

The low baseline severity and relatively high personal social functioning suggest patients’ remitted states at the beginning of dose tapering. Durations of illness ranged widely from 0.8 to 35 years (mean 9.6 ± 8.5 years), and the majority had a history of psychiatric hospitalization (56.9%) and a history of relapse (66.7%). Baseline treatment comprised 10 oral and 2 long-acting injectable antipsychotics with dose ranges skewed to the lower end of each agent, with an average CPZE dose of around 200 mg/d (Table 1 and details in Supplementary Figure S1). Distribution of different antipsychotics was scattered across all subgroups, while oral-form aripiprazole (*n* = 14) was the most prevalent, followed by a tie between olanzapine and risperidone (both *n* = 8). Statistically significant differences were found between the four subgroups in diagnosis of schizophrenia (versus other schizophrenia-spectrum psychotic disorders), history of relapse, and baseline symptom severity (Table 2).

### Changes of clinical characteristics after 2-year follow-up

By the end of 2-year follow-up, SR patients on average reduced 58.5% of baseline doses and stayed well under an average post-reduction dose CPZE 89.6 ± 79.7 mg/d, while statistically significant improvement was seen in symptom severity, clinical global impression, personal social functioning, and subjective quality of life. More SR patients were also able to have a full-time job. No such significant changes were seen in CR patients and FR patients (Table 2).

### Factors associated with different dose-tapering patterns

Univariate logistic regression analyses of factors associated with each trajectory revealed that a history of relapse was significantly associated with the category of conservative reducer (odds ratio (OR): 4.64, 95% confidence interval (CI): 1.25–17.25, *p* = 0.022) (Table 3). Besides, diagnosis of schizophrenia was marginally related to increased likelihood of being a conservative reducer (OR: 4.63, 95% CI: 0.87–24.52, *p* = 0.071) yet decreased likelihood of being a sequential reducer (OR: 0.28, 95% CI: 0.07–1.16, *p* = 0.078), while all 6 FR patients were with the diagnosis of schizophrenia. Results were examined further using multivariate logistic regression analysis with backward stepwise selection to see if any model provided additional information (Table 4).

**Table 3.** Univariate logistic regression analyses for factors related to designated trajectories

	SR			CR			FR		
	Coefficient	95% CI	*p* value	Coefficient	95% CI	*p* value	Coefficient	95% CI	p value
Age	0.022	−0.039 to 0.083	0.479	0.037	−0.023 to 0.097	0.227	−0.109	−0.230 to 0.011	0.076
Gender (Male)	−0.634	−1.803 to 0.535	0.288	1.041	−0.096 to 2.179	0.073	0.044	−1.659 to 1.748	0.959
Employed	0.241	−0.951 to 1.434	0.692	−0.513	−1.680 to 0.655	0.390	0.693	−1.023 to 2.409	0.429
Diagnosis, schizo	−1.288	−2.721 to 0.145	0.078	1.533	−0.134 to 3.199	0.071	–^a^	–	–
DOI (years)	0.023	−0.044 to 0.091	0.499	0.017	−0.049 to 0.083	0.617	−0.118	−0.293 to 0.058	0.188
Hx of admission	0.270	−0.900 to 1.439	0.651	0.113	−0.998 to 1.224	0.842	−1.099	−2.898 to 0.700	0.231
Hx of relapse	<0.001	−1.218 to 1.218	1.000	**1.535**	**0.223** to **2.848**	**0.022**	−1.594	−3.410 to 0.222	0.085
CPZE mg/d	0.0005	−0.0038 to 0.0048	0.804	−0.0001	−0.004 to 0.004	0.966	−.0047	−0.013 to 0.003	0.261
PANSS	−0.068	−0.154 to 0.019	0.126	−0.016	−0.076 to 0.043	0.587	0.069	−0.004 to 0.142	0.065
CGI-S	−0.097	−0.823 to 0.629	0.794	−0.245	−0.945 to 0.455	0.492	−0.071	−1.147 to 1.005	0.897
PSP	0.031	−0.041 to 0.104	0.395	0.015	−0.052 to 0.081	0.669	−0.020	−0.120 to 0.080	0.691
EQ-5D-VAS	0.016	−0.030 to 0.063	0.491	−0.023	−0.068 to 0.021	0.305	0.043	−0.034 to 0.119	0.275
MSQ	−0.106	−0.562 to 0.351	0.651	0.122	−0.322 to 0.564	0.590	0.170	−0.541 to 0.881	0.640

Abbreviations: *Subgroup naming:* SR: sequential reducer; CR: conservative reducer, comprised by MR, AR and BR; FR: failed reducer; *Baseline and clinical variables:* BL: baseline; CGI-S: clinical global impression-severity; CPZE chlorpromazine equivalent dose; EQ-5D-VAS: EuroQoL-5D visual analogue scale; FU: end of follow-up status; PANSS: positive and negative syndrome scale; PSP: personal and social performance; SD: standard deviation; SE: standard error.

a All 6 FR patients were with the diagnosis of schizophrenia. Statistics with *p* values < 0.05 were displayed in bold.

**Table 4.** Multivariate logistic regression analyses for factors related to designated trajectories

	SR			CR			FR		
	Odds ratio	95% CI	*p* value	Odds ratio	95% CI	*p* value	Odds ratio	95% CI	*p* value
Age	–	–	–	–	–	–	0.765	0.565 to 1.036	0.084
Gender (male)	–	–	–	–	–	–	–	–	–
Full–time Employed, Yes	–	–	–	**0.146**	**0.025** to **0.841**	**0.031**	–	–	–
Diagnosis, (schizophrenia)	0.260	0.053 to 1.281	0.098	6.595	0.921 to 47.20	0.060	19.62	0.386 to 998	0.138
Duration of illness (years)	–	–	–	–	–	–	–	–	–
History of admission, Yes	–	–	–	–	–	–	–	–	–
History of relapse, Yes	–	–	–	**11.20**	**2.012** to **62.37**	**0.006**	–	**–**	**–**
CPZE mg/d	–	–	–	–	–	–	0.985	0.970 to 1.001	0.071
PANSS	0.917	0.832 to 1.010	0.077	–	–	**–**	1.282	0.966 to 1.702	0.085
CGI–S	–	–	–	0.396	0.138 to 1.134	0.084	–	–	–
PSP	–	–	–	–	–	–	1.302	0.890 to 1.904	0.174
EQ-5D-VAS	1.040	0.980 to 1.105	0.197	0.955	0.900 to 1.013	0.128	1.096	0.976 to 1.230	0.120
MSQ	0.626	0.3336 to 1.167	0.140	–	–	–	–	–	–
LR χ^2^		df (4):8.83			df (5):17.07			df (6):18.15	
*P* > chi2		0.066			**0.004**			**0.006**	
Pseudo *R*^2^		0.133			0.242			0.491	

Abbreviations: *Subgroup naming:* SR: sequential reducer; CR: conservative reducer, comprising MR, AR and BR; FR: failed reducer; *Baseline and clinical variables:* BL: baseline; CGI-S: clinical global impression-severity; CPZE chlorpromazine equivalent dose; EQ-5D-VAS: EuroQoL-5D visual analogue scale; FU: end of follow-up status; PANSS: positive and negative syndrome scale; PSP: personal and social performance; SD: standard deviation; SE: standard error.

Statistics with *p* values < 0.05 were displayed in bold.

Multivariate analysis showed that the relationship between history of relapse and being a conservative reducer was more pronounced (OR: 11.20, 95% CI: 2.012–62.37, *p* = 0.006). Moreover, patients with a full-time job at baseline were significantly less likely to become conservative reducers (OR: 0.146, 95% CI: 0.03–0.84, *p* = 0.031). In the multivariate model, the factors of having a history of relapse, and not having a full-time job, together with a diagnosis of schizophrenia, lower clinical global severity and lower quality of life, was significantly predictive of becoming conservative reducers (likelihood ratio (LR) χ^2^, degree of freedom (df) (5): 17.07; *p* = 0.004; Pseudo *R*^2^: 0.242).

Only 6 failed reducers were identified in this cohort, all with a diagnosis of schizophrenia yet no other variable was found to be significantly associated with relapse during dose tapering. Multivariate logistic regression analyses showed that the model composed of younger age, lower baseline antipsychotic dose, higher baseline PANSS score, higher personal social functioning, better quality of life, and having a full-time job was significantly associated with relapse during drug tapering (LR χ^2^, df (6):18.15; *p* = 0.006; Pseudo *R*^2^ = 0.4913).

Univariate logistic regression also failed to find any demographic or clinical variable with a significant impact on the chance of being a sequential reducer. The model with the lowest probability as checked by LR Chi2, composed of a lower baseline PANSS score, lower medication satisfaction, with a diagnosis of other schizophrenia-spectrum psychotic disorder, and higher quality of life, was still not significantly predictive for indicating a sequential reducer (LR χ^2^, df (4):8.83; *p* = 0.065; Pseudo *R*^2^ = 0.133).

## Discussion

This study is one of the few to depict dose-tapering trajectories for stable patients receiving antipsychotic maintenance treatment [19, 31, 32]. Our results suggest that it is a long and winding road to attempt discontinuation of antipsychotics. Based on a study design that empowered patients to participate in shared decision-making when eligible to take further dose reduction, or to re-escalate dose to the previous level once having suspected signs of relapse, results of this study can serve as valuable references to studies and practice in a real-world setting [47].

Even though the algorithm in this study guided clinicians to taper antipsychotics very slowly and in a hyperbolic manner as suggested previously [42], only 1 out of 3 patients (18/51) was able to reduce a quarter of the current dose consecutively every 6 months, repeating it four times in 2 years, without experiencing a relapse. The average post-reduction dose of these SR patients, CPZE 90 mg/d, is much lower than the dose, 200 mg/d, identified to be an important factor associated with successful dose reduction by a meta-analysis [23]. SR patients reached a significantly improved condition in many aspects of life, instilling hope for patients remitted from psychosis to pursue such desirable outcomes. However, no clinical variable was identified that helps to predict who would have a better chance to reach this ideal trajectory. Thus, individualized patient care appears to be the key to successful dose-tapering. Also, a sound support system is an important protective factor that was not formally recorded as a covariate in this study, and it deserves better attention in future studies.

The relatively low relapse rate of this cohort, not higher than their maintenance counterparts [41], may be a result of many patients (24/51) bearing a cautious and conservative stance towards dose tapering. They either opted to stay at a slower pace (MR), or re-escalate to a higher dose whenever unsure if warning signs were re-emerging (AR or BR), as to prevent a full-blown relapse. Such a cautious attitude correlates highly with the fact that 5 out of 6 CR patients have a history of relapse during previous treatment. Patients in this subgroup on average reduced 28% of baseline dose in 2 years (40, 29, and 0% for MR, AR, and BR, respectively), while no substantial improvements in symptoms and quality of life were attained (Table 2). This result may raise questions about whether it is worth investing good efforts for tapering doses with such a modest gain. Nevertheless, as long as they could maintain stable remission, in the future there is still a chance to reduce the dose by tapering a smaller dose at a time and allowing longer observation if suspected re-emerging warning signs will wane without re-escalating the dose.

Although only a small number of patients experienced relapse during dose tapering, several factors for precaution were identified. In addition to the diagnosis of schizophrenia, a well-known risk factor of relapse [6], the higher baseline PANSS score suggests symptom stability is still an important prerequisite when considering dose tapering. Interestingly, higher personal social functioning, better quality of life, and having a full-time job were linked to increased risks of relapse, suggesting that these patients might overlook the risk of relapse because they had been doing quite well at the entry of the study. Indeed, two patients with good functioning and quality of life confessed that they had stopped taking antipsychotics completely on their own accord, leading to a relapse soon after their discontinuation attempts. Additionally, a lower baseline antipsychotic dose is likely to contribute to a higher risk of relapse, which is in accord with the hyperbolic dose–response curves of antipsychotic dose and relapse prevention which illustrates a disproportionately increased risk of relapse at the lower dose end [48], as well as the prediction of Horowitz et al. that it will be easier to taper a larger fraction of medication at higher dose levels, while it will be safer to only cut off a much smaller fraction of dose at the lower dose level [42]. This phenomenon deserves better attention as it will be crucial if wishing to discontinue antipsychotics completely with a favorable ending.

As the sample size of each subgroup is small and not feasible for sophisticated statistical analyses, this study was more exploratory than confirmatory, so several limitations must be addressed. First, most participants were cooperative with this delicate tapering procedure, implying that they were in a better position to anticipate a good individual prognosis, thus the results may not be generalizable to patients who were not able to follow the guided processes reliably. Second, most patients had more than one psychotic episode, a condition in which most guidelines do not usually recommend discontinuing antipsychotic medications [25]. Thus, as indicated previously [8], not only patients but also their doctors were more likely to adopt a cautious attitude during dose tapering, which may inflate the proportion of patients categorized in a conservative group. Third, to minimize the risk of relapse, the algorithm defined the threshold of relapse as having symptoms aggravated for more than 1 week. However, this may be too sensitive to capture those who were able to accommodate antipsychotic-associated withdrawal symptoms and regain stabilization without an increased dose after 1 week [49], again resulting in an exaggerated proportion of patients staying with a conservative trajectory yet preventing them to reduce more doses which might be achievable via a more flexible tapering approach [50, 51]. Lastly, the relatively small sample sizes in each pattern did not allow sophisticated statistical analyses; three trajectories even had to be collapsed into one subgroup (CR), while subtle yet important differences may exist among MR, AR, and BR patients.

Echoing the advocacy of Marder and Zito: “We encourage a sense of curiosity about the possibility of dose reduction and discontinuation in appropriate patients.” [52], as well as the advocacy of Murray and Di Forti: “Guidelines need to be developed on when and how slowly to reduce antipsychotics, and in whom it is appropriate to eventually stop them.” [53], this report employs a visual presentation to recognize and define the patterns of dose-tapering trajectories along a zigzag path. The proposed naming provides a framework to depict individual patient’s course. To develop guidelines for delivering better clinical practice to this patient population, researchers can adopt such an intuitive descriptor system, examine the progress of patient’s dose reduction with a clear view portrayed by reasonable anchor points, and compare findings based on a common ground. Also, clinicians can discuss with their remitted patients by referring to the visual illustration of dose-tapering processes as well as highlighting the pros, cons, and warnings during shared decision-making. Currently, three-fourths of this cohort agreed to receive follow-up by our research team and we will monitor their trajectories and outcomes via this lens in the longer term.

## Supporting information

Liu et al. supplementary material Liu et al. supplementary material

Liu et al. supplementary material Liu et al. supplementary material

## Data Availability

The data that support the findings of this study are available on request from the corresponding author.

## Supplementary material

The supplementary material for this article can be found at https://doi.org/10.1192/j.eurpsy.2023.2440.

## References

[1] Perkins DO, Gu H, Weiden PJ, McEvoy JP, Hamer RM, Lieberman JA. Predictors of treatment discontinuation and medication nonadherence in patients recovering from a first episode of schizophrenia, schizophreniform disorder, or schizoaffective disorder: a randomized, double-blind, flexible-dose, multicenter study. J Clin Psychiatry. 2008;69(1):106–13. 18312044 10.4088/jcp.v69n0114

[2] Correll CU, Rubio JM, Kane JM. What is the risk-benefit ratio of long-term antipsychotic treatment in people with schizophrenia? World Psychiatry. 2018;17(2):149–60. 29856543 10.1002/wps.20516 PMC5980517

[3] Hui CL, Honer WG, Lee EH, Chang WC, Chan SK, Chen ES, et al. Predicting first-episode psychosis patients who will never relapse over 10 years. Psychol Med. 2019;49(13):2206–14. 30375301 10.1017/S0033291718003070

[4] Takeuchi H, Siu C, Remington G, Fervaha G, Zipursky RB, Foussias G, et al. Does relapse contribute to treatment resistance? Antipsychotic response in first- vs. second-episode schizophrenia. Neuropsychopharmacology. 2019;44(6):1036–42. 30514883 10.1038/s41386-018-0278-3 PMC6462044

[5] Alvarez-Jimenez M, Priede A, Hetrick SE, Bendall S, Killackey E, Parker AG, et al. Risk factors for relapse following treatment for first episode psychosis: a systematic review and meta-analysis of longitudinal studies. Schizophr Res. 2012;139(1–3):116–28. 22658527 10.1016/j.schres.2012.05.007

[6] Bowtell M, Ratheesh A, McGorry P, Killackey E, O’Donoghue B. Clinical and demographic predictors of continuing remission or relapse following discontinuation of antipsychotic medication after a first episode of psychosis. A systematic review. Schizophr Res. 2018;197:9–18. 29146020 10.1016/j.schres.2017.11.010

[7] Thompson A, Singh S, Birchwood M. Views of early psychosis clinicians on discontinuation of antipsychotic medication following symptom remission in first episode psychosis. Early Interv Psychiatry. 2016;10(4):355–61. 25962446 10.1111/eip.12244

[8] Yen K, Liu CC, Lin YT, Chien YL, Hsieh MH, Liu CM, et al. Discontinuing antipsychotic medication after remission from first-episode psychosis: a survey of psychiatrists’ attitudes in Taiwan. Neuropsychiatr Dis Treat. 2022;18:465–75. 35261544 10.2147/NDT.S339866 PMC8898187

[9] Hui CL, Wong AK, Leung WW, Lee EH, Chan SK, Chang WC, et al. Psychiatrists’ opinion towards medication discontinuation in remitted first-episode psychosis: a multi-site study of the Asian network for early psychosis. Early Interv Psychiatry. 2019;13(6):1329–37. 30485671 10.1111/eip.12765

[10] Hui CLM, Honer WG, Lee EHM, Chang WC, Chan SKW, Chen ESM, et al. Long-term effects of discontinuation from antipsychotic maintenance following first-episode schizophrenia and related disorders: a 10 year follow-up of a randomised, double-blind trial. Lancet Psychiatry. 2018;5(5):432–42. 29551618 10.1016/S2215-0366(18)30090-7

[11] Tiihonen J, Tanskanen A, Taipale H. 20-year nationwide follow-up study on discontinuation of antipsychotic treatment in first-episode schizophrenia. Am J Psychiatry. 2018;175:765. 29621900 10.1176/appi.ajp.2018.17091001

[12] Fusar-Poli P, Smieskova R, Kempton MJ, Ho BC, Andreasen NC, Borgwardt S. Progressive brain changes in schizophrenia related to antipsychotic treatment? A meta-analysis of longitudinal MRI studies. Neurosci Biobehav Rev. 2013;37(8):1680–91. 23769814 10.1016/j.neubiorev.2013.06.001 PMC3964856

[13] Ho BC, Andreasen NC, Ziebell S, Pierson R, Magnotta V. Long-term antipsychotic treatment and brain volumes: a longitudinal study of first-episode schizophrenia. Arch Gen Psychiatry. 2011;68(2):128–37. 21300943 10.1001/archgenpsychiatry.2010.199 PMC3476840

[14] Voineskos AN, Mulsant BH, Dickie EW, Neufeld NH, Rothschild AJ, Whyte EM, et al. Effects of antipsychotic medication on brain structure in patients with major depressive disorder and psychotic features: neuroimaging findings in the context of a randomized placebo-controlled clinical trial. JAMA Psychiatry. 2020;77(7):1–11. 10.1001/jamapsychiatry.2020.0036 PMC7330722 32101271

[15] Vita A, De Peri L, Deste G, Barlati S, Sacchetti E. The effect of antipsychotic treatment on cortical gray matter changes in schizophrenia: does the class matter? A meta-analysis and meta-regression of longitudinal magnetic resonance imaging studies. Biol Psychiatry. 2015;78(6):403–12. 25802081 10.1016/j.biopsych.2015.02.008

[16] Liu CC, Takeuchi H. Achieving the lowest effective antipsychotic dose for patients with remitted psychosis: a proposed guided dose-reduction algorithm. CNS Drugs. 2020;34(2):117–26. 31741178 10.1007/s40263-019-00682-8

[17] Ozawa C, Bies RR, Pillai N, Suzuki T, Mimura M, Uchida H. Model-guided antipsychotic dose reduction in schizophrenia: a pilot, single-blind randomized controlled trial. J Clin Psychopharmacol. 2019;39(4):329–35. 31188232 10.1097/JCP.0000000000001046

[18] Zhou Y, Li G, Li D, Cui H, Ning Y. Dose reduction of risperidone and olanzapine can improve cognitive function and negative symptoms in stable schizophrenic patients: a single-blinded, 52-week, randomized controlled study. J Psychopharmacol. 2018;32(5):524–32. 29493377 10.1177/0269881118756062

[19] Huhn M, Leucht C, Rothe P, Dold M, Heres S, Bornschein S, et al. Reducing antipsychotic drugs in stable patients with chronic schizophrenia or schizoaffective disorder: a randomized controlled pilot trial. Eur Arch Psychiatry Clin Neurosci. 2021;271(2):293–302. 32062728 10.1007/s00406-020-01109-y PMC7960583

[20] Tsuboi T, Suzuki T, Bies RR, Remington G, Pollock BG, Mimura M, et al. Challenging the need for sustained blockade of dopamine D(2) receptor estimated from antipsychotic plasma levels in the maintenance treatment of schizophrenia: a single-blind, randomized, controlled study. Schizophr Res. 2015;164(1–3):149–54. 25864950 10.1016/j.schres.2015.03.025

[21] Graff-Guerrero A, Rajji TK, Mulsant BH, Nakajima S, Caravaggio F, Suzuki T, et al. Evaluation of antipsychotic dose reduction in late-life schizophrenia: a prospective dopamine D2/3 receptor occupancy study. JAMA Psychiatry. 2015;72(9):927–34. 26131622 10.1001/jamapsychiatry.2015.0891

[22] Uchida H, Suzuki T, Graff-Guerrero A, Mulsant BH, Pollock BG, Arenovich T, et al. Therapeutic window for striatal dopamine D2/3 receptor occupancy in older patients with schizophrenia: a pilot PET study. Am J Geriatr Psychiatry. 2014;22:1007–16. 25217025 10.1016/j.jagp.2013.01.045

[23] Tani H, Takasu S, Uchida H, Suzuki T, Mimura M, Takeuchi H. Factors associated with successful antipsychotic dose reduction in schizophrenia: a systematic review of prospective clinical trials and meta-analysis of randomized controlled trials. Neuropsychopharmacology. 2020;45(5):887–901. 31770770 10.1038/s41386-019-0573-7 PMC7075912

[24] Rodolico A, Siafis S, Bighelli I, Samara MT, Hansen WP, Salomone S, et al. Antipsychotic dose reduction compared to dose continuation for people with schizophrenia. Cochrane Database Syst Rev. 2022;11(11):Cd014384. 36420692 10.1002/14651858.CD014384.pub2 PMC9685497

[25] Shimomura Y, Kikuchi Y, Suzuki T, Uchida H, Mimura M, Takeuchi H. Antipsychotic treatment in the maintenance phase of schizophrenia: an updated systematic review of the guidelines and algorithms. Schizophr Res. 2020;215:8–16. 31784340 10.1016/j.schres.2019.09.013

[26] Wils RS, Gotfredsen DR, Hjorthoj C, Austin SF, Albert N, Secher RG, et al. Antipsychotic medication and remission of psychotic symptoms 10years after a first-episode psychosis. Schizophr Res. 2017;182:42–8. 28277310 10.1016/j.schres.2016.10.030

[27] Wunderink L, Nieboer RM, Wiersma D, Sytema S, Nienhuis FJ. Recovery in remitted first-episode psychosis at 7 years of follow-up of an early dose reduction/discontinuation or maintenance treatment strategy: long-term follow-up of a 2-year randomized clinical trial. JAMA Psychiatry. 2013;70(9):913–20. 23824214 10.1001/jamapsychiatry.2013.19

[28] Harrow M, Jobe TH, Faull RN. Do all schizophrenia patients need antipsychotic treatment continuously throughout their lifetime? A 20-year longitudinal study. Psychol Med. 2012;42(10):2145–55. 22340278 10.1017/S0033291712000220

[29] Chouinard G, Samaha AN, Chouinard VA, Peretti CS, Kanahara N, Takase M, et al. Antipsychotic-induced dopamine supersensitivity psychosis: pharmacology, criteria, and therapy. Psychother Psychosom. 2017;86(4):189–219. 28647739 10.1159/000477313

[30] Horowitz MA, Murray RM, Taylor D. Confounding of antipsychotic discontinuation studies by withdrawal-related relapse. Schizophr Bull. 2022;48(2):294–5. 34964477 10.1093/schbul/sbab146 PMC8886601

[31] Wunderink L, Nienhuis FJ, Sytema S, Slooff CJ, Knegtering R, Wiersma D. Guided discontinuation versus maintenance treatment in remitted first-episode psychosis: relapse rates and functional outcome. J Clin Psychiatry. 2007;68(5):654–61. 17503973 10.4088/jcp.v68n0502

[32] Gaebel W, Stricker J, Riesbeck M. The long-term antipsychotic treatment of schizophrenia: a selective review of clinical guidelines and clinical case examples. Schizophr Res. 2020;225:4–14. 31806527 10.1016/j.schres.2019.10.049

[33] Moncrieff J, Lewis G, Freemantle N, Johnson S, Barnes TRE, Morant N, et al. Randomised controlled trial of gradual antipsychotic reduction and discontinuation in people with schizophrenia and related disorders: the RADAR trial (research into antipsychotic discontinuation and reduction). BMJ Open. 2019;9(11):e030912. 10.1136/bmjopen-2019-030912 PMC6887002 31780589

[34] Sturup AE, Jensen HD, Dolmer S, Birk M, Albert N, Nielsen M, et al. TAILOR - tapered discontinuation versus maintenance therapy of antipsychotic medication in patients with newly diagnosed schizophrenia or persistent delusional disorder in remission of psychotic symptoms: study protocol for a randomized clinical trial. Trials. 2017;18(1):445. 28962668 10.1186/s13063-017-2172-4 PMC5622425

[35] Weller A, Gleeson J, Alvarez-Jimenez M, McGorry P, Nelson B, Allott K, et al. Can antipsychotic dose reduction lead to better functional recovery in first-episode psychosis? A randomized controlled-trial of antipsychotic dose reduction. The reduce trial: study protocol. Early Interv Psychiatry. 2019;13:1345–56. 30488637 10.1111/eip.12769

[36] Begemann MJH, Thompson IA, Veling W, Gangadin SS, Geraets CNW, van ’t Hag E, et al. To continue or not to continue? Antipsychotic medication maintenance versus dose-reduction/discontinuation in first episode psychosis: HAMLETT, a pragmatic multicenter single-blind randomized controlled trial. Trials. 2020;21(1):147. 32033579 10.1186/s13063-019-3822-5 PMC7006112

[37] Liu CC, Hsieh MH, Chien YL, Liu CM, Lin YT, Hwang TJ, et al. Protocol of guided antipsychotic reduction to reach minimum effective dose (GARMED) in patients with remitted psychosis based on pragmatic design. Early Interv Psychiatry. 2022;16(2):178–85. 33751764 10.1111/eip.13144

[38] Liu CC, Liu CM, Chien YL, Lin YT, Hsieh MH, Hwang TJ, et al. Challenging the minimum effective antipsychotic dose during maintenance: implications from 10-year follow-up of first episode psychosis. Front Psychiatry. 2021;12:714878. 34557119 10.3389/fpsyt.2021.714878 PMC8453020

[39] Leucht S, Samara M, Heres S, Davis JM. Dose equivalents for antipsychotic drugs: the DDD method. Schizophr Bull. 2016;42(Suppl 1):S90–4. 27460622 10.1093/schbul/sbv167 PMC4960429

[40] Woods SW. Chlorpromazine equivalent doses for the newer atypical antipsychotics. J Clin Psychiatry. 2003;64(6):663–7. 12823080 10.4088/jcp.v64n0607

[41] Liu CC, Hsieh MH, Chien YL, Liu CM, Lin YT, Hwang TJ, et al. Guided antipsychotic reduction to reach minimum effective dose (GARMED) in patients with remitted psychosis: a 2-year randomized controlled trial with a naturalistic cohort. Psychol Med. 2023:1–9. 10.1017/S0033291723000429 PMC10719630 36896797

[42] Horowitz MA, Jauhar S, Natesan S, Murray RM, Taylor D. A method for tapering antipsychotic treatment that may minimize the risk of relapse. Schizophr Bull. 2021;47(4):1116–29. 33754644 10.1093/schbul/sbab017 PMC8266572

[43] Cheng JJ, Ho H, Chang CJ, Lane SY, Hwu HG. Positive and negative syndrome scale (PANSS): establishment and reliability study of a mandarin Chinese language version. Chin J Psychiatry. 1996;10:251–8.

[44] Wu BJ, Lin CH, Tseng HF, Liu WM, Chen WC, Huang LS, et al. Validation of the Taiwanese mandarin version of the personal and social performance scale in a sample of 655 stable schizophrenic patients. Schizophr Res. 2013;146(1–3):34–9. 23478156 10.1016/j.schres.2013.01.036

[45] Vernon MK, Revicki DA, Awad AG, Dirani R, Panish J, Canuso CM, et al. Psychometric evaluation of the medication satisfaction questionnaire (MSQ) to assess satisfaction with antipsychotic medication among schizophrenia patients. Schizophr Res. 2010;118(1–3):271–8. 20172695 10.1016/j.schres.2010.01.021

[46] Chang LR, Lin YH, Chang HC, Chen YZ, Huang WL, Liu CM, et al. Psychopathology, rehospitalization and quality of life among patients with schizophrenia under home care case management in Taiwan. J Formos Med Assoc. 2013;112(4):208–15. 23537867 10.1016/j.jfma.2012.01.018

[47] Relton C, Torgerson D, O’Cathain A, Nicholl J. Rethinking pragmatic randomised controlled trials: introducing the “cohort multiple randomised controlled trial” design. BMJ. 2010;340:c1066. 20304934 10.1136/bmj.c1066

[48] Leucht S, Bauer S, Siafis S, Hamza T, Wu H, Schneider-Thoma J, et al. Examination of dosing of antipsychotic drugs for relapse prevention in patients with stable schizophrenia: a meta-analysis. JAMA Psychiatry. 2021;78(11):1238–48. 34406325 10.1001/jamapsychiatry.2021.2130 PMC8374744

[49] Horowitz MA, Murray RM, Taylor D. Withdrawal-associated relapse is a potential source of bias. Lancet Psychiatry. 2021;8(9):747–8. 34419175 10.1016/S2215-0366(21)00250-9

[50] Horowitz MA, Moncrieff J, de Haan L, Bogers J, Gangadin SS, Kikkert M, et al. Tapering antipsychotic medication: practical considerations. Psychol Med. 2022;52(1):32–5. 34542027 10.1017/S0033291721003299

[51] Horowitz MA, Taylor D. How to reduce and stop psychiatric medication. Eur Neuropsychopharmacol. 2022;55:4–7. 34688998 10.1016/j.euroneuro.2021.10.001

[52] Marder SR, Zito MF. Will I need to take these medications for the rest of my life?. World Psychiatry. 2018;17(2):165–6. 29856554 10.1002/wps.20519 PMC5980574

[53] Murray RM, Di Forti M. Increasing expectations and knowledge require a more subtle use of prophylactic antipsychotics. World Psychiatry. 2018;17(2):161–2. 29856571 10.1002/wps.20517 PMC5980442

