# Supplementary material for: Dose-tapering trajectories in patients with remitted psychosis undergoing guided antipsychotic reduction to reach minimum effective dose
Source: Eur Psychiatry. 2023 Aug 14;66(1):e66. doi: 10.1192/j.eurpsy.2023.2440 (PMC10594210; doi:10.1192/j.eurpsy.2023.2440)
Supplement: Liu et al. supplementary material [file S0924933823024409sup001.doc]

**Supplemental** **Figure 1. Trajectories of individual dose tapering**

Sequential Reducers (SR): those who could taper doses at each designated timepoint successfully for 4 steps in 2 years


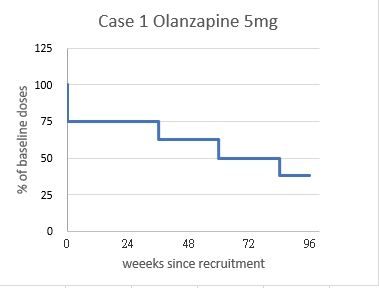

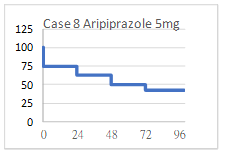

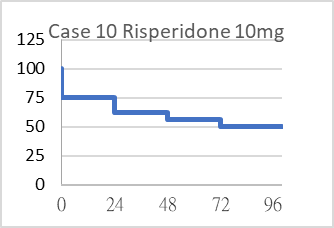

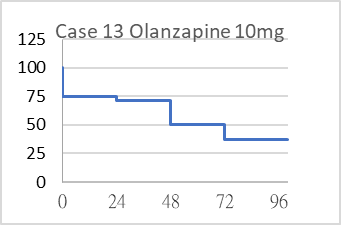

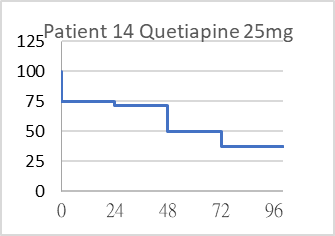


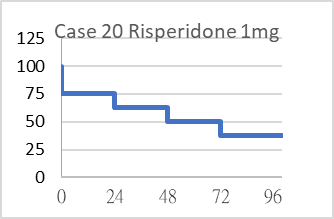

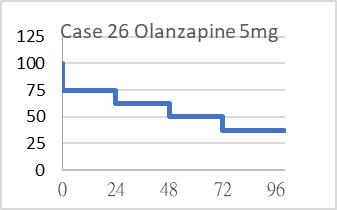

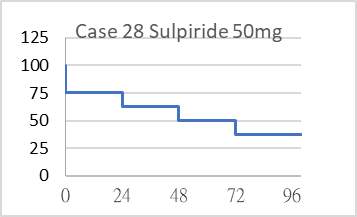

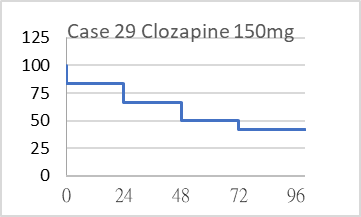

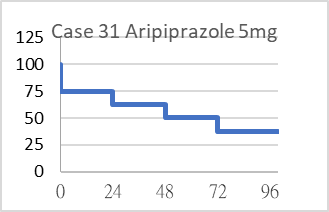


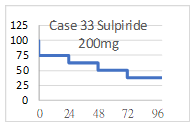

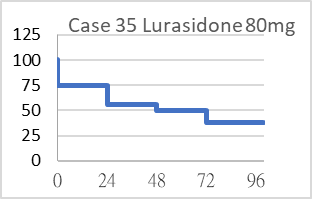

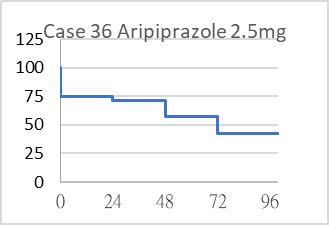

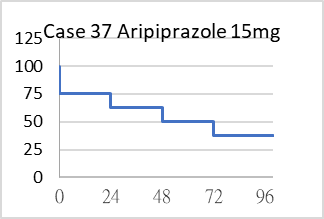

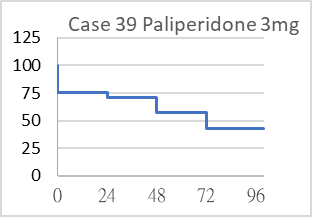


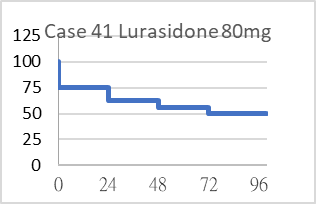

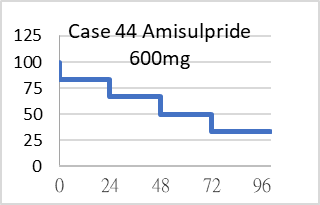

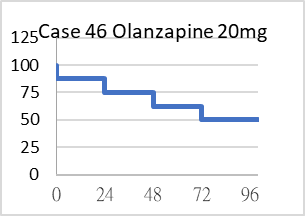


The vertical axis is the % of baseline dose; the horizontal axis is weeks since dose tapering.

Modest Reducers (MR): those who took a cautious pace in tapering, reduced 1 to 3 steps, with no re-escalation to previous step, during 2 years


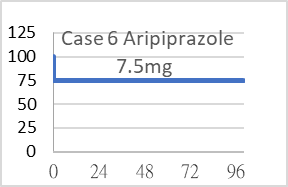

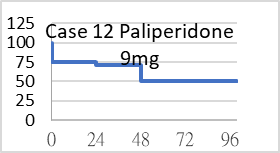

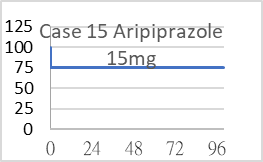

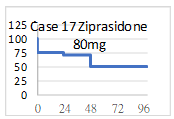

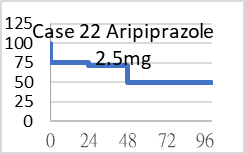


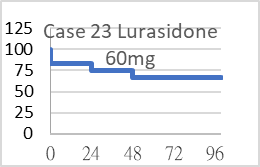

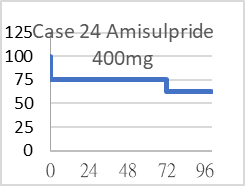

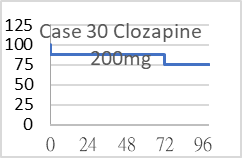

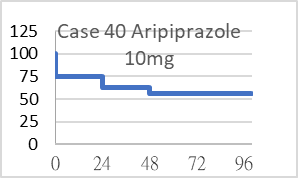

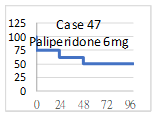


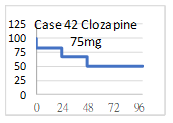

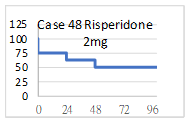

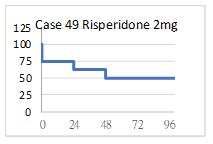


Alert Reducers (AR): those who have had reduced at least 2 steps yet re-escalated dose back to the level between their baseline dose and the lowest dose they have reached during the course of dose tapering


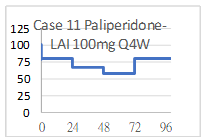

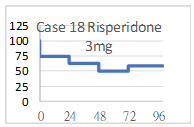

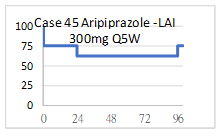


Baseline Returners (BR): those who have tapered at least 1 step but returned to baseline dose for concern about risks of relapse and were able to stay in remission throughout the course


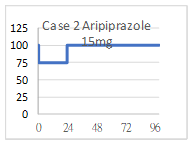

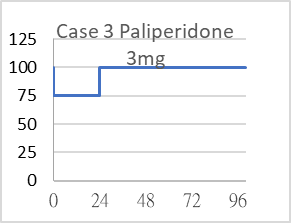

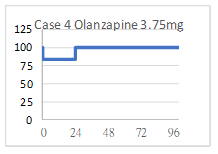

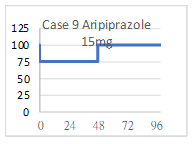

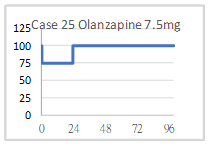

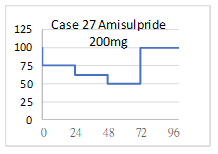

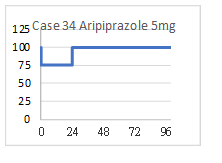


Failed Reducers (FR): those who had a relapse during dose tapering and were in need of a dose higher than their baseline level


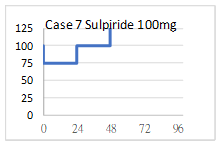

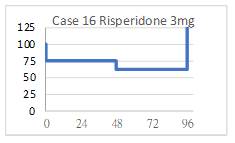

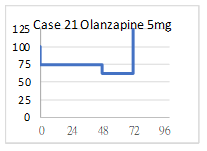

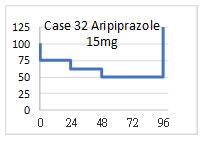

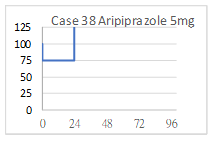

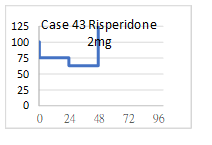


Early Exits (EE): those who left the trial after tapering at least 1 step without relapse before the end of 2-year follow-up.


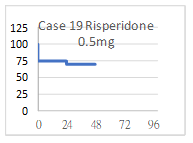

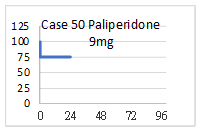

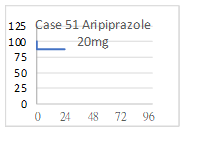


**Supplemental Table 1. Eligible criteria**

| **Inclusion criteria:** ^a^  1. Both male and female outpatients or patients at psychiatric daycare service  2. Age 18–60 years old at the time of screening  3. A diagnosis of schizophrenia, schizophreniform disorder, psychosis NOS, based on the DSM-5 criteria  4. With a Positive and Negative Syndrome Scale (PANSS), score < 3 in all 3 positive symptoms (P1: delusion, P2: conceptual disorganization, P3: hallucination) and 2 general symptoms (G9: unusual thought, G5: mannerism and posturing) for at least 3 months  5. With a PANSS score < 4 in all 3 negative symptoms (N1: blunted affect, N4: social withdrawal, N6: lack of spontaneity/flow in conversation) for at least 3 months ^b^  6. Currently receiving antipsychotic treatment at a fixed dose for at least 3 months, including long-acting injectable antipsychotic ^d^  7. A second antipsychotic agent only used for a low-dose, as needed adjuvant purpose ^c^  8. No revised use of benzodiazepines, antidepressants, anticholinergics, or other concomitant medications during the past 3 months |
| --- |
| **Exclusion criteria:**  1. A score of 5 or more on any of the 30 PANSS rating items at screening  2. Admission to the acute psychiatric unit during the past 6 months  3. A change in dose of current antipsychotic medication in recent 3 months  4. Concomitant use of mood stabilizers, such as lithium, valproic acid, or other anti-epileptic drugs  5. Mental retardation known as IQ below 70 prior to the diagnosis of schizophrenia  6. A history of pervasive mental disorder or bipolar disorder  7. A medical condition with significant cognitive sequelae  8. A history of substance dependence during the past 6 months  9. Currently in pregnancy or breastfeeding |
| a. Patients with multi-episode psychosis were recruited because many of them tried dose reduction/discontinuation by themselves even though usually not recommended to do so.  b. We also broadened Andreasen et al.’s remission criteria (2005) to include patients with higher scores in negative symptoms (< 4 rather than < 3), as negative symptoms might not respond to antipsychotics as much as positive and general symptoms, while excessive dopamine D2 blockade might be associated with impaired cognition; thus a trial of dose reduction should be justifiable to this group of patients.  c. The most commonly used antipsychotics for this purpose are chlorpromazine 50 mg or quetiapine 25 mg. These agents would stay at the same dose during observation.  d. In this cohort all participants were on a stable dose for at least six months. |

**Supplemental Table 2.**

|  |  | FR* (n=4/49) |  |  | FR (n=6/51) |  |
| --- | --- | --- | --- | --- | --- | --- |
|  | Coefficient | 95% CI | P value | Coefficient | 95% CI | P value |
| Age | -0.138 | -0.299~0.024 | 0.094 | -0.109 | -0.230~0.011 | 0.076 |
| Gender (Male) | 0.044 | -2.001~2.090 | 0.966 | 0.044 | -1.659~1.748 | 0.959 |
| Employed | 0.693 | -1.362~2.749 | 0.509 | 0.693 | -1.023~2.409 | 0.429 |
| Diagnosis, schizo | - | - | - | - | - | - |
| DOI (years) | -0.079 | -0.256~0.099 | 0.385 | -0.118 | -0.293~0.058 | 0.188 |
| Hx of admission | -0.405 | -2.454~1.643 | 0.698 | -1.099 | -2.898~0.700 | 0.231 |
| Hx of relapse | -1.999 | -4.353~0.354 | 0.096 | -1.594 | -3.410~0.222 | 0.085 |
| CPZE mg/d | -0.007 | -.0191~0.004 | 0.207 | -.0047 | -0.013~0.003 | 0.261 |
| PANSS | 0.053 | -0.056~0.161 | 0.340 | 0.069 | -0.004~0.142 | 0.065 |
| CGI-S | -0.073 | -1.380~1.235 | 0.913 | -0.071 | -1.147~1.005 | 0.897 |
| PSP | 0.012 | -0.122~0.146 | 0.857 | -0.020 | -0.120~0.080 | 0.691 |
| EQ-5D-VAS | -0.008 | -0.090~0.075 | 0.857 | 0.043 | -0.034~0.119 | 0.275 |
| MSQ | 0.004 | -0.813~0.820 | 0.993 | 0.170 | -0.541~0.881 | 0.640 |

* The two patients with good functioning and quality of life confessed that they had stopped taking antipsychotics completely on their own accord which led to a relapse soon after their discontinuation attempts were excluded in this analysis.
